# Foundation models for bioinformatics

**DOI:** 10.1002/qub2.69

**Published:** 2024-07-24

**Authors:** Ziyu Chen, Lin Wei, Ge Gao

**Affiliations:** ^1^ State Key Laboratory of Protein and Plant Gene Research School of Life Sciences Biomedical Pioneering Innovative Center (BIOPIC) & Beijing Advanced Innovation Center for Genomics (ICG) Center for Bioinformatics (CBI) Peking University Beijing China; ^2^ Changping Laboratory Beijing China

**Keywords:** ChatGPT, foundation models, large language models, transformer

## Abstract

Transformer‐based foundation models such as ChatGPTs have revolutionized our daily life and affected many fields including bioinformatics. In this perspective, we first discuss about the direct application of textual foundation models on bioinformatics tasks, focusing on how to make the most out of canonical large language models and mitigate their inherent flaws. Meanwhile, we go through the transformer‐based, bioinformatics‐tailored foundation models for both sequence and non‐sequence data. In particular, we envision the further development directions as well as challenges for bioinformatics foundation models.

## 1 INTRODUCTION

**FIGURE 1:**
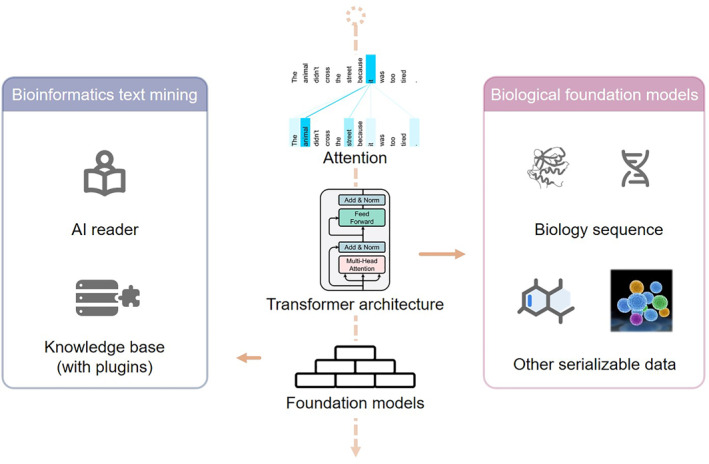
Applications of foundation models for bioinformatics. Textual foundation models, represented by large language models, can help with bioinformatics text mining, functioning as an AI reader or knowledge base with plugins. Transformer architecture can also be adapted for biological sequences and other serializable data to build large‐scale biological foundation models. Part of the illustration was adapted from Refs [3, 4].

Deep learning is undergoing a paradigm shift with the boom of large‐scale foundation models pre‐trained on a large corpus of data and adapted to multiple downstream tasks [1, 2]. The foundation nature of these models not only highlights their broad applications but also implies their incompleteness. Here, we will focus on two topics: canonical textual large language models (LLMs) for text‐based bioinformatics data mining and foundation models adapted for biological data (Figure 1).

Most, if not all, foundation models are based on transformer architecture [3]. The key concept, “attention”, behind transformer, which emphasizes inter‐token relationship, first came into public sight in an “additive attention” setup over a bi‐LSTM network [5]. Later in 2017, attention was further switched to a “dot‐product/multiplicative attention” setup, which was named transformer [3], for high scalability up to billions of parameters, enabling parallel pre‐training on a massive corpus of unlabeled data in a self‐supervised manner. The pre‐training strategy mainly goes into two categories: encoder‐only, BERT‐like [6, 7] architecture which takes an auto‐encoding mask language model training objective and decoder‐only, GPT‐like [8, 9] architecture which takes an autoregressive causal language model loss.

After chat‐oriented fine‐tuning and alignment [10, 11], these pre‐trained models become canonical textual LLMs we now come across in daily life, including ChatGPTs, Claude, Gemini, as well as LLaMA and BLOOM family [12, 13]. The biological and biomedical information mining has been widely adopted as a direct downstream application of these LLMs. Meanwhile, transformer architecture has been adapted to biological sequences such as DNA, RNA, proteins, and serializable data such as single‐cell omics and small molecules, benefiting from the scalability and capacity of transformer.

## 2 TEXTUAL LLMs FOR BIOINFORMATICS TEXT MINING

LLMs’ inherent logical reasoning capabilities [14], rooted from their abilities to extract syntactic and semantic structure of input text [15], turn LLMs into an effective “AI reader” for context‐sensitive summarizing across a large corpus of literature [16]. These abilities enable LLMs to participate in the traditional manual curation process of databases. Furthermore, LLMs with further contrastive fine‐tuning can generate semantic feature vector representations of the given sentences [17, 18] (also see OpenAI’s embedding application programming interfaces (APIs) [19]), extending their abilities from key‐word‐based searching to semantics‐based searching. Meanwhile, LLMs can be regarded as a “knowledge base” about biological concepts [20]. Several works test the performance of LLMs on biological question answering, including gene interactions, biological pathways [21], genomic functions [22], and marker‐gene‐based cell type annotation [23]. In particular, it has been well demonstrated that biomedical‐tailored LLMs trained over medical literature are able to better understand and answer domain‐specific questions [24, 25, 26].

Of note, the fact that current LLMs suffer from hallucination (which is defined as “generating syntactically and semantically correct but unfaithful or nonsensical text” [27]) makes them good copilots, other than reliable advisers, for bioinformatics workflows. For instance, ChatGPT is queried for additional participants of the Circadian Clock pathway in a Reactome curation trial run [28]. ChatGPT proposed 13 candidates, among which 7 have literature supports but are overlooked in traditional manual curation, 5 cannot be confirmed, and 1 is inaccurate.

Several strategies have been shown to improve LLMs’ real‐world usability.

1. Prompt tuning is always your first resort when coming into problems with LLMs. Previous work [29] demonstrated the importance of role‐prompting, chain‐of‐thought prompting, and in‐context learning in gene relationship mining and promoted an iterative prompt refinement strategy to boost performance.
2. It is well known that LLMs are prone to hallucination and cannot tell what is unknown, especially when asked for detailed questions or questions out of the knowledge base of the training corpus [27]. Though the popular LLMs’ pre‐training corpus includes several biological databases, such as PubMed abstracts and PubMed Central full texts, incorporating more biological texts into the pre‐training process may still be needed to mitigate hallucination in biology‐related tasks.
3. Retrieval augmented generation (RAG) [30, 31] has been suggested for suppressing hallucination. The key idea behind RAG is to provide extra information, which is retrieved by text‐embedding‐based database search, directly into the chat context of LLMs. The reasoning process of LLMs can leverage this information for better handling of downstream tasks.
4. More generally, as modern chatbots such as ChatGPTs have been pre‐trained to understand APIs and return in JavaScript Object Notation mode, they know when and how to call functions or tools provided as plugins to strengthen themselves. Additional abilities can be injected into LLMs by these plugin functions to support vector database searching for RAG, web browsing, and PubMed searching, etc. [32]. These plugins and growing model scale may further increase the abilities of LLMs to handle biological tasks.

## 3 FOUNDATION MODELS FOR BIOLOGICAL DATA

Next, we move on to discuss about foundation models pre‐trained on biological data, including biological sequence data (DNA, RNA, and proteins) and data that is serializable (small molecules and single cell omics).

Transformer architecture is naturally suitable for biological sequences. There are several key choices when designing a foundation model for sequence data. (1) Whether we should choose a BERT‐like structure, which excels at extracting meaningful embeddings, or a GPT‐like structure that harnesses the generation abilities. Briefly, for BERT‐like models, representations learned from large‐scale pre‐training data are contextualized representation for each token (i.e., amino acid or nucleotide) and are to some extent a replacement for multi‐sequence alignment (MSA) [33, 34]. The contextualized representation can be further employed for multiple downstream tasks including structure prediction, mutation effects inference, and functional properties prediction (see more details for protein [34, 35, 36, 37], DNA [38, 39, 40, 41, 42], and RNA [43, 44]). Oppositely, GPT‐like models can be adapted for control tag‐based *de novo* protein generation or protein engineering tasks with high diversity and success rate [45, 46, 47, 48, 49]. (2) Whether to pre‐train at a single sequence or the MSA level [50]. (3) The scale we tokenize and model sequences: DNABERT‐2 [41] utilizes Byte‐Pair Encoding algorithms to tokenize DNA sequences and treats multiple residues as one token to improve compute efficiency, while ESM All‐Atom [51] chooses to expand the residue representation to the atom level during pre‐training for finer grain modeling and incorporates the small molecule modality.

Just like transformers’ application in computer vision [52, 53], it can also be adapted to other serializable biological data. Small molecules can be easily serialized as SMILES strings and fed into transformer to learn representations to predict molecular properties [54, 55], drug‐target interaction [56], and other functional tasks, thus promoting drug design. Serialization and application of transformer architecture are more challenging for single‐cell omics [57]. Geneformer [58], GeneCompass [59], and Nicheformer [60] serialize the single‐cell count matrix by only taking the relative ranking of the normalized expression of individual genes into consideration. Meanwhile, scBERT [61], scGPT [62], scFoundation [63], and xTrimoGene [64] replace the position embedding in classic transformer models into gene embedding and regard binned or transformed expression as tokens. Contextualized gene‐level embeddings, cell‐level embeddings, and attention patterns from the pre‐trained models can be tailored for downstream tasks, including cell type annotation [58, 61, 62], perturbation analysis [62, 64], regulatory network inference [58, 62] etc.

Another line of work introduces textual LLMs as external knowledge for single‐cell analysis. GenePT [65] and scELMo [66] utilize textual LLMs‐based embeddings as an orthogonal approach to aforementioned expression‐based embeddings for downstream tasks. As it has been demonstrated that LLMs can generate semantic embeddings of sentences, these studies provide text summaries of genes and cells to textual LLMs to generate textual gene/cell embeddings and aggregate them based on expression profiles to generate cell embeddings for downstream tasks. These studies pose a new direction of integrating textual and biology foundation models to improve performance, usability, and interpretability.

## 4 DISCUSSION AND PERSPECTIVE

Up till now, foundation models based on transformer still suffer from several limitations. Interpretability was initially assumed to be an advantage of transformer architecture, given that “attention” should highlight inter‐relationship inside sequences. However, later works find that there are still gaps between interpretation and “attention” [67], and additional steps are needed [68]. Though the transformer operation itself can be applied on the sequence of any length if not taking position embedding into account, its space and time complexity grow quadratically with the length of sequence and consequently limits the input context length. This field is calling for a new generation of drop‐in replacement for transformer operators without hurting its scalability and O(1) information path length between long‐range dependencies in the network [3]. This object may be achieved by (1) better implementation handling memory bounding [69], (2) sparse or low rank approximation [70, 71, 72], and (3) utilizing state space models or some other forms [42, 73, 74, 75, 76].

Further development of foundation models for bioinformatics points out several directions.

1. The capability boundary of textual LLMs on more biological information retrieval tasks needs further testing and can be augmented with plugins to LLMs.
2. In particular, as the field has learned “the bitter lesson” [77] that scaling law [78, 79] is the secret of success of LLMs nowadays, foundation models extending to billions of parameters for biological data remain to be tested. And more research is needed to answer what emergent properties [80] are in this field. Growing model size in turn presses the need for growing volume of well‐cleaned open‐source biological data.
3. Taking inspirations from the success of multimodal models across texts, images, and voices, multimodal models across different kinds of biological sequences, different single cell omics, biomedical images and omics, are also interesting topics and attract growing attention.

## AUTHOR CONTRIBUTIONS

**Ziyu Chen**: Investigation; visualization; writing – original draft. **Lin Wei**: Writing – review & editing. **Ge Gao**: Project administration; supervision.

## CONFLICT OF INTEREST STATEMENT

The authors declare that they have no conflict of interest.

## ETHICS STATEMENT

This article is a perspective article and does not contain any studies with human or animal subjects performed by any of the authors.
